# How Can We Prevent False Hydrophobia?†Read under the title of “The Attitude of Legal Medicine *versus* Hydrophobia,” before the Society of Medical Jurisprudence and State Medicine, at the Hall of the Academy of Medicine, June 10th, 1886.

**Published:** 1886-07

**Authors:** E. C. Spitzka

**Affiliations:** Consulting Neurologist to the North Eastern Dispensary; Attending Physician Department of Nervous Diseases of the German Poliklinik; W. & S. Tuke Prize Essayist


### Art. XIII.—HOW CAN WE PREVENT FALSE HYDROPHOBIA? †

BY E. C. SPITZKA, M.D,

*Consulting Neurologist to the North Eastern Dispensary; Attending Physician Department of Nervous Diseases of the German Poliklinik; W. & S. Tuke Prize Essayist.*

Gentlemen :—If compelled to apologize at the outset, for the fact that the preamble of my paper is so lengthy, as compared with its conclusion, I think I can justly refer to the obscurity which still enshrouds the subject of hydrophobia, in palliation. I do not believe that there is another branch of inquiry, in which time-honored tradition, erroneous observation, ill-balanced reasoning, and immature theory play so large a part as in that relating to the transmission of rabies from animals to man. If I deal more with the question of spurious than with real hydrophobia, it is because my experience with the former is considerable, and with the latter infinitely small; indeed I would say *nil*, but that I wish to avoid discussing a question both whose sides are represented by able thinkers and observers, whether there really is such a thing as genuine hydrophobia in man, due to transmission from lower animals *and to nothing else!*

In view of the length of the preliminary portion of the paper I think it well to announce its propositions and problems at

† Read under the title of "The Attitude of Legal Medicine *versus* Hydrophobia," before the Society of Medical Jurisprudence and State Medicine, at the Hall of the Academy of Medicine, June 10th, 1886.

the outset, as then, my aim in the body of the paper may appear more clear :—

I. There is, at present, no proven epidemic of hydrophobia either in New York city or in its vicinity.

II. A number of deaths have occured from spurious hydrophobia, (lyssophobia), in New York city and its vicinity, during the past nine months.

III. The agitation of the subject of Pasteur's method of preventive inoculation, for hydrophobia, and the accompanying strained accounts of suffering and death from that disease, are responsible for these deaths.

IV. To prevent the serious and oft-times fatal imaginary disease, it is necessary to inculcate a healthy public sentiment, which shall frown down the agitation of questions, which are of a strictly scientific character, by persons who are neither biologists nor physicians, and who have no other motive than the creation of a sensation, regardless of consequences.

V. It should be strictly inculcated on all officers entrusted with the preservation of the public peace and public health, that the only way to determine whether a dog is suffering from rabies or no, is to submit him to inspection by competent veterinarians in a living condition. The desirability of some such ordinance is attested by the fact that persons on the road to death from hydrophobia, have recovered on learning that the dog who bit them remained alive and well.

VI. The results of researches on hydrophobia, should, for the present, remain within the domain of technical literature. There are so many problems connected with the question of rabies, which are as yet, and promise to remain for some time, unsettled, that it would be tantamount to criminal recklessness to publish prematurely alarming discoveries in the lay press.

VII. The method of demonstrating rabies by direct inoculation of the brain is fallacious : The conclusion drawn by Liautard, from an experiment thus performed, that the Riverdale dog was mad, was obtained by misleading methods.

VIII. The means to be adopted to prevent future outbreaks of spurious hydrophobia, is to muzzle the dogs, to dam up the torrents of ink flowing from the pens of hasty investigators within appropriate receptacles, and to exclude sensational publications from the household.

Probably the most seriously misunderstood and misrepresented members of our community, at the present moment, are the various representatives of the species *canis familiaris.* When a journalistic hydrophobia becomes established, these unfortunate beings are not allowed the wretched privilege of falling ill of any other disease than the one which it has pleased some magnate of the press to declare epidemic. If a dog does not eat, he has hydrophobia; if he draws his tail between his legs, beware! if his eyes have a strange expression, or, what is the same thing, if you think they have, shoot him on the spot! If he resents your fooling with him, get your doctor to open a subscription for you and start for Pasteur's laboratory by the next steamer, at reduced rates! Hereafter, canine pathology will prove an easy science to master. It will consist of but one simple syllogism, as follows:— All symptoms of canine disease are symptoms of hydrophobia; all dogs presenting such symptoms should be destroyed; hence, for all sick dogs there is but the remedy of the bullet or the policeman's club. Am I exaggerating? Have you followed up the history of the so-called epidemic since the needless, wanton and wicked agitation of the matter was begun a few months ago? Do you remember that for a week after the first flaming announcement was made relative to the Newark epidemic, several scores of cases of hydrophobia were reported from every part of the country? Do you recollect a single case of the kind in which any attempt, meriting the adjective scientific—nay, any attempt whatever, was made to verify the existence of that dreaded disease and where it really was verified? Allow me to briefly reproduce a few of the cases which were published far and wide, with the deliberate and malicious purpose of causing a sensation. I will show you later that the result was the production of imaginary disease, real alarm, real suffering, and something else, which was but too real:—Death!

On Ninth avenue a horse was seized with some nervous symptoms. It was reported that the animal had been bitten by a dog. There was some contradiction on this head, but it made no difference. An alarming announcement of an outbreak of hydrophobia in this city was published in the largest possible type. The ink was scarcely dry, before a competent veterinarian decided that the horse was suffering from an ordinary equine disease, known as cerebro-spinal meningitis. Another epidemic was next announced from Ironton, with this heading: "The Mad Dog Scare; Citizens Arousing Themselves." The nucleus for this report was a fit occuring in a little dog, due to his having swallowed more chicken bones than he could digest, and this the autopsy showed. An epidemic of hydrophobia was also reported from Southern Illinois; several persons were represented as having died. The Ninth report of the Illinois State Board of Health, in speaking of this epidemic embodies the conclusion that it was spurious, and to show this cites the following instance in point. Wm Bundschuh, of Unith, Alexander County, was severely bitten a year ago, by a dog who was summarily executed. The wound healed very slowly, and the patient lived in a dread of hydrophobia which was greatly increased when a certain newspaper furnished column after column of Pasteur's pronunciamentos. About November, last, he became afflicted with fever, had convulsions, and was pronounced hydrophobic, by a physician who duly chloroformed him. The patient's brother-in-law calling in Dr. Royal, of Villa Ridge, the latter discovered that it was a case of severe intermittent fever, and under appropriate treatment, succeeded in master-both the real and the imaginary affection, and thus saved Bundschuh's life.

A similar case of spurious hydrophobia, occured in the practice of Dr. Feeny, of Staten Island. The patient was firmly told that he had an ordinary form of delirium, and ultimately recovered. I believe if this patient or his Illinois fellow-sufferer had been starved, chloroformed and terrified, the verdict would have been "died from the bite of a mad dog." You are all acquainted with at least a part of the history of the so-called Newark epidemic. Out of the mass of imperfectly collected testimony, the following facts may be regarded as approximately true: A dog had bitten a number of other dogs as well as six children. The transgressor was killed, in accordance with the time-honored custom of hanging such criminals first, and trying them afterwards. Four of the children were sent to Pasteur, two remained in Newark, and Mr. Runge, a sensible veterinarian, had the bitten dogs locked up where they could do no harm on the one hand, but on the other, where it could be determined whether they really had been inoculated with hydrophobia. After keeping these dogs under careful daily observation for four months, Mr. Runge discharged them from custody as "not guilty." Meanwhile, the Pasteurized children returned from Paris, to figure in a dime museum, and to such extent as they constituted a source of income to their parents, were better off than those which were not Pasteurized. The latter, however, were of far greater value to scientific men, than the four who were exposed to the gaze of morbid sight-seers. They also remained free from hydrophobia, and showed, that in this case at least, the alarm had been a false one, and that a great deal of public anxiety had been wantonly provoked, and philanthropic gush as fruitlessly expended. Had this agitation no worse results in Newark? What did the pound-keeper, George Neall die of? I am aware that able and learned colleagues pronounced him to be suffering from hydrophobia. I do not intend to widen the scope of this paper by taking up the views of those who deny the existence of such a disease in the human being. I simply take the account as I find it. It seems that for several days, that is, during the entire time of his illness, Neall obtained no drink, and ate nothing except an apple. He received hypodermic injections of chloral, atropin, and, had he lived longer, curare, cocaine and vapor baths would have been tried, That he was under the dominion of a terrible fear, is evident from the fact that *he got up shortly before his death, walked into the kitchen, placed his hand in a pail of water and had a spasm in consequence, which lasted half an hour.* Were starvation, depressing neurotic medicines, and terror, without an effect on this patient's vitality?

I received the following interesting and valuable account of the Arlington case from Dr. J. A. Exton, President of the Hudson County Medical Society.

Willie Klee, aet 10, an inmate of the Sacred Heart Industrial School was bitten by a dog which was chained January 17th, in the City of Paterson, N. J., while home on a vacation. He returned to the Industrial School January 18th, and was to all appearances in his usual health.

January 22d, he was with the others of the institution successfully vaccinated, notwithstanding his arm bore evidence of former successful vaccination. January 23d, he was sent to bed by the sisters because he had *fits*. In giving me a history of the case, they said he would growl and bite, or make attempts to do so; that saliva would flow from his mouth and that he seemed in constant fear that some one was going to harm him. January 24th, I saw him at one o'clock, he looked bright and intelligent, pulse 72, temperature normal. The sisters in charge said he had passed a comfortable night after having taken 10 grains Potass Bromid. but that at five in the morning, when the bell was sounded, he immediately went into a convulsion which lasted some five minutes, during which he growled and barked, etc.

Desiring to get móre knowledge upon the case I ordered the bell to be sounded. The lad was napping when this was done, and immediately he slowly raised himself in the bed, snapped, barked audibly many times, growled, clutched the covers spasmodically and hid his head and moaned, the arms and legs remaining fixed. I allowed him to sleep some time, when I carefully roused him, and taking some water turned it from one glass to another in front of him, and purposely dropping some upon him. This caused no seizure. and upon suggesting to drink some, he consented and drank with a relish. I ordered him a vapor bath to be continued twenty to thirty minutes, before beginning which he was to have ½ ʒ Fl. Extract Jaborandi, this dose to be repeated every half hour until three were taken. After coming from the bath he was to have and did have one teaspoonful of the following

℞ Potass Bromid.

Hydrat Chloral aa ʒid.

Aqua P—ʒii.

M. Sig. Coch 1 in water or syrup every three hours unless asleep, and all noises which were thought to annoy him carefully discontinued.

Upon my visit next day the boy was perfectly quiet and had been so since coming from the vapor bath.

The bell, which on the previous day caused a seizure, was again tried, but to no purpose. He had eaten properly and without the least difficulty of deglutition and was in every way, so far as I could see, himself again.

The Jaborandi of course caused free salivation, which fixed the case in the minds of the attendants as one of Rabies.

Question ? Was this Hydrophobia or was it Hysteria.

The great influence which expectant attention has in provoking peculiar sensations, even in calm and courageous persons, is illustrated in the case of J—— C——, of Watertown, now under the treatment of my friend, Dr. J. D. Spencer. In this case, the patient, if not a disbeliever in hydrophobia, was like his physician convinced that the vast majority of cases in man were of hysterical origin. I quote his own statement, as made in response to the question, what medical treatment had been instituted in his case?*

"What has been my treatment ? Dr. Spencer must tell you that. I have followed his advice and taken his medicine ; have kept cool and calm ; saw no one but the doctor for 11 days. My symptoms have been carefully noted down, sometimes by myself and sometimes by my wife. I noticed alarm in the

***New York Times* of June 2, 1886.

doctor's face the eighth day. My temperature was normal and my pulse 120. I calmed myself as best I could. That morning I began to have sharp pains in my eyes. The eyeballs enlarged so that I could see to read nonpareil without glasses, something that I had not done for twelve years. There was an uneasy restlessness all day. Although my temper is usually calm, it was with the utmost endeavor that I could control myself. I wanted to scold or hit somebody, and chewed up a whole lead pencil in my fury. I retired between 7 and 8 in the evening, not because I was sleepy, but for the purpose of getting out of the way of everybody. Hot and cold flashes passed through my system until midnight, while I tossed and tumbled about the bed wrapped in flannel sheets. With almost lightning suddenness then began pains, sharp ones, seemingly coming from the wound at first, but so rapid that within a short time they seemed to start from every part of my system. I was burning, in pain, and thirsty. My first impulse was to take a run in the cool air, my second was to stay in bed and fight it out. I did not care to call any one, fearing that their talk would cause me to lose what little reason I had. I felt so light that it seemed to me there was danger of my floating out of bed; I firmly grasped the side of the bed and held on, believing the unusual pain and heat would soon pass away. I held myself in that position until a few minutes before 4—nearly four hours—when the pain and heat suddenly stopped. My mind was perfectly clear. The flannel sheets were wet with perspiration, I got up terribly exhausted and attempted to take a teaspoonful of aromatic spirits of ammonia in half a glass of water which had been prepared for me to drink through the night. My stomach repelled it with terrible force. I waited until the stomach had quieted down, and then I swallowed the medicine quickly, shutting my mouth with a grim determination that it should not come out. The stomach acted the same as before, but I would not open my mouth. It was hard, and my throat did not feel just right. Well, I got it down, and got such a terrible wrenching that I have not yet recovered. I took my medicine next day with my eyes closed. There were some of the symptoms of the night before, but they were lighter, and have now passed away entirely—at least, I think so. I could easily have gone crazy had not I kept my senses when burning with pain and thirst; would probably have done so had not Dr. Spencer sent me home from the office to remain quiet. I have a sore leg, and just as soon as that is healed I will be at work again, satisfied that there is as much hydrophobia in a person's mind as in a dog's bite, and believing that our best American physicians can cure what disease there is if patients will only control themselves and not go off into hysterics.

I believe no one here, whatever apprehension he might entertain as to the future prospects of this case, would call the symptoms of Mr. C—— above detailed, symptoms of hydrophobia.

Dogs become mad; insane, in other words, as men do, from various causes and in various ways. I had hoped to exhibit an insane cat, to-night, the pet of a friend, which had been shot in the head, and which, fortunately did not run across the path of any "alarmist." In Japan, as Prof. Law informs us, the boys produce what is supposed to be rabies, and indeed resembles traditional rabies in every respect, by administering to dogs a fungus,* which grows on pine trees. So great is the resemblance of the counterfeit to the original, that the dogs are killed by way of precaution, and it is claimed that the artificial affection can be transmitted by the bite. If that be true, it is fortunate that the human being is not a biting animal, for they have the habit in Siberia of drinking a decoction of a fungus which produces intoxication, and sometimes delirium. When the fungus is scarce the urine of those intoxicated is consumed by others desirous of participating in the same enjoyment with the aristocratic first imbiber of the *muscarin.* As yet we have had no opportunity of learning whether the latter might not confer a share of his pleasures through biting his poorer fellow consumers.

On the 4th of February, William Higgins shot himself from fear of hydrophobia. He had been bitten by a dog six months before. The Sunday before his death, while he was on a visit to his sister in Sixth street, he said he had carefully studied the preliminary symptoms of the disease from the papers and he detected them in himself, and told her not to be alarmed if she heard of his death. †

The following horrible case, I submit in the original language of the report; it requires no comment:

"Cohoes, N. Y., February 3d.‡—Sunday night George Waterhouse, aged 14, was taken ill and showed signs of hydrophobia He grew rapidly worse, and last night was in such a suffering condition, with eyes protruding and mouth frothing, that the physicians saw there was no hope of saving him, and to end his sufferings *ordered that he be smothered,* which was done. Waterhouse was bitten by a dog *six years* ago, while a resident of Lansingburg. The wound healed rapidly at the time and caused no inconvenience until now."

I believe the number of persons dying of so called hydrophobia almost simultaneously with the agitation of the subject of Pasteur's methods, but who had been bitten between nine

---

* Bukeryo, according to a communication from his student, Mr. Sâzé. (*Pepper's System of Medicine by American Authors*, vol. I., p. 891).

† *New York World*, February 5, 1886.

‡Probably the *Times* correspondence of February 4th, 1886, the scrap is unfortunately not marked.

months and nine years previous will be found to reach a hundred or more.*

Let us see what one of the leading American comparative pathologists says regarding such occurrences:

"But all such cases of prolonged incubation in man are at the least extremely doubtful. Man often contracts a pseudo hydrophobia as the result of fear, and is curable by moral suasion only; and as no such protracted incubations are noticed in the lower animals, and as no one of these abnormally deferred attacks in man has been verified by successful innoculations on animals, it is prudent to reserve a full assent until they are supported by better testimony. A specimen of such cases is that recorded by Chirac, in which a cadet bitten at Montpelier, afterwards spent ten years in Holland, and then, returning and hearing that his fellow-cadet, bitten by the same dog, had died of hydrophobia, he also manifested the disease and died."

The ablest review which has yet been made of the subject of spurious hydrophobia is by Dulles, of Philadelphia. He shows that this condition may result from 1. Disorders of the stomach, especially in very young children, as well as from inflammation of the œsophagus and stomach in adults.*† 2. Disorders of the respiratory apparatus. Their resemblance in many of their symptoms is so great that, in 1625, Aromatarius devoted a monograph to proving that hydrophobia is nothing

---

*Case by Dujardin-Beaumetz (*British Medical Journal* letter of January 15th, 1886)—The patient had been bitten in the right hand, in 1884, by a dog *that did not seem to labor under any rabic symptoms*, but was nevertheless killed. The doctor—I say, most unwisely!—expressed a fear of hydrophobia on the pâtient's complaining of pain at the site of the wound on November 1st, 1885. The sequel can be imagined. Pasteur as usnal made a theatrical declamation, "I can preserve, but I do not cure," and the patient then expired in terrible agony. A Major Morley died about the same time at Malta. twelve months after a bite from his own dog, which is said to have been seized with rabies at Dover.

† Disorders mistaken for Hydrophobia. Transactions of the Medical Society of the State of Pennsylvania for 1884.

‡ He cites Prichard's description of so called intestinal mania as follows: "The mouth and fauces if examined, generally present a diseased aspect. The fauces and velum pendulum are red; the vessels injected covered in patches with mucus. . . . . The mouth is viscid, and the patient generally spits out a frothy slime in all directions. . . . . There is an ardent thirst. , . . . In many cases the patient has an aversion to all food. . . . . The skin is clammy and cold. . . . . The complexion is often flushed, the eyes wild, glassy, with a superabundant lachrymal secretion, the tunica conjunctiva is not unfrequently injected with blood, the patient is scarcely tolerant of light, . . . . The urine is scanty and high-colored. . . . . The pulse is rapid and irritable."

but a contagious sore throat.* 3. Disorders of the circulatory apparatus: that inflammatory conditions of the heart and its coverings give rise to the spmptoms of hydrophobia is a fact well recognized in most treatises and special articles on the subject. 4. Systemic conditions: such are rheumatic fever, gout (H. Sutton), uraemia, intermittent fever, mercurialization† and pregnancy.‡ 5. Disorders of the nervous system: meningitis, pachymeningitis, tumors and parasites of the brain, simple neuritis, acute mania, deliria, § alcoholism, tetanus, epileptic delirium, hysteria, and narcotic poisoning.

I am able to add three instances to those collected by Dr. Dulles, where symptoms commonly or erroneously regarded as hydrophobia were present. The first of these was a case I saw in Williamsburgh with Dr. Fuhs. I published it in the *New York Medical Record.*‖ It was one of epileptic delirium, in which the patient snapped continually at everything about him with an appearance of grim ferocity, and developed terrible laryngeal spasms with growling noises in addition. I remember, as Dr. Fuhs accompanied me a short distance in

* Dulles sustains this view for some cases, "although by a strange process of inversion the striking inflammatory lesions which have been found in them *post mortem* have been looked upon as effects, when, in fact, they were really causes." He cites a case reported by Vidal, where undoubted laryngitis was mistaken for hydrophobia.

† Dr. Reid, in 1817, mentions a young woman dying of hydrophobia caused by mercury taken for syphilis. It was really a case of ulcerative stomatitis.

‡ Mazars de Cazelles saw hydrophobia occurring eleven times during the first four months of pregnancy in the same woman. Doleris mentions a like occurrence.

§ Westphal (*Archiv fur Psychiatrie II.*, p. 520) says that acute delirium is often confounded with hydrophobia, and that, in depressed and hypochondrical subjects, the bite of a healthy dog will cause hydrophobia

‖ The aspect of the patient was truly horrible, with a ghastly grin he opened and shut his jaws with such intensity that one feared his teeth must crack; at the same time he tried to catch the hands of those restraining him with his jaws, and, actually—although in epileptic unconsciousness—attempted to discover where these hands were, by using his tongue as a feeler. When his tongue felt a hand, his jaws would snap at it. His eyes rolled, the pupils were widely dilated, and he caught at his (contracted) protruding larynx with such a vigorous clutch, that, had he not been prevented, he would have torn it out.

the street, his saying : "Many a hydrophobia scare has been started on a less sufficient basis"—and he was right. The second case I saw through the courtesy of Dr. T. A. McBride. While walking through the wards of the Presbyterian Hospital he was notified that a man had arrived with the diagnosis of hydrophobia. He was in a close apartment ; as I walked in at Dr. M's invitation, the mouse-nest odor of typhus fever was apparent ; as the physician lifted up the patient's clothing a beautiful exanthema was displayed. In his delirium the poor fellow had spoken of dogs,* and his medical attendant had thence made the diagnosis of hydrophobia. It was stated to me later that the autopsy revealed the existence of epidemic meningitis. The third case I cite from a foreign journal.† In this case, one of disseminated sclerosis, described by Pollak, there was a remarkable horror of water. The patient, a weak-minded child, suffered from polyuria, yet it was impossible to get it to take water, and almost impossible to force down a few drops of other liquids, such as wine, beef tea, or lemonade. Even when it made efforts to swallow them, they were regurgitated convulsively, the entire body being agitated by tremors. Finally the mere sight of these fluids caused it the greatest distress. For fourteen months this singular dread of fluids continued ; at the end of that time the child died. The reporter of this interesting case attributes the hydrophobia to the sclerotic changes which were found in the oblongata.

The resemblance between the spurious hydrophobia and the so-called real affection is so great that I cannot criticise anyone for believing, with Dulles, that the existence of a genuine hydrophobia in man is not proven. In order to avoid citing *ex parte* evidence, I subjoin some extracts from "Pepper's System of Medicine by American Authors," the particular author being a believer in the reality of hydrophobia :

"The languor fever and nervousness attendant in extreme heat tend not

* Dogs play a prominent part in the delusions of the insane. Kiernan, who has made several contributions to the subject of spurious hydrophobia (*Am. Journal of Neurology and Psychiatry*, May, 1883), mentions a Katatonic patient, who was "followed to church by droves of dogs."

†Congenitale, multiple Herdsclerose des Central-nervensystems ; partieller Balkenmangel von Doctor Ladislaus Pollak.—*Archiv fur Psychia trie XII.*, p. 157. (Congenital multiple sclerosis, in foci, with partial defect of the corpus callosum).

only to hasten the activity of any disease-germs actually present in the system ; but also strongly favor the increase of that nervous fear which *so often generates a fatal pseudo-hydrophobia* (lyssophobia) *in persons that have been bitten by dogs.*"*

† "The most difficult to distinguish from the genuine disease are those cases in which hydrophobia occurs as a disease of the imagination, the result of fear—the lyssophobia or *hydrophobie non-rabique* of the writers. In these there is always the history of a bite, the cicatrix even may have become the seat of congestive redness, itching or neuralgic pains, and these, acting on a susceptible brain, develop a disease which is hardly distinguishable from true hydrophobia, and which is quite as fatal if left to run its course. These cases have usually less reflex susceptibility than genuine hydrophobia; the attack mostly occurs shortly after some conversation on the subject, and especially about the effects of the bites on others, and the victim is seen to have a nervous organization, and may even be known to have been subject to hysteria or other nervous disorders. *At the same time, the concentration of the mind on this subject sometimes produces even structural changes in the medulla.*‡ and the reflex susceptibility in co-ordination with the other symptoms may be *almost perfect*. In a case reported a few years ago, by Hammond, the symptoms appeared perfectly characteristic, and at the necropsy, circumscribed points of congestion were found near the roots of the vagus ; yet the dog that bit this man was said to be alive and well, and in the absence of any successful inoculation from biter or bitten, the case must be presumed to have been lyssophobia."

"Many cases with a more favorable issue are recorded. Bellenger had a patient who had been bitten by his cat, and manifested violent paroxysms of hydrophobia, but was instantly cured by the sight of the animal in good health. Brouardel records that a man was bitten by his dog which afterwards disappeared. He was seized with severe hydrophobia which continued for two days: when the lost dog was found and presented to him the symptoms disappeared. Trousseau speaks of a magistrate whose hand had been licked by his hound, which immediately after attacked a flock of sheep, so that many of them died of rabies. The master then manifested hydrophobia, but as death was deferred beyond the usual time he concluded it was not genuine and recovered. Prof. Dick was called to visit a man who had been bitten by a favorite dog, which, while suffering from distemper, had manifested severe hydrophobic symptoms, and had been given up by the attending physicians. He succeeded in convincing the subject that as the dog had had distemper, and as no two great diseases could exist in the same system, it could not have had rabies. In spite of the false premises, this reasoning had the desired effect, and the patient recovered. A few years ago a boy twelve years old, in Ithaca, N. Y., was bitten by a dog supposed to be rabid, and in due time manifested hydrophobia, which advanced rapidly, until he was having a violent paroxysm every half hour, and it was pronounced impossible for him to survive another day. At this time I saw him, observed

---

*Op cit, p. 888.

† Op. cit. p. 901.

‡ Law states no authority or special observation to justify the statement beyond the case mentioned.

that he had a nervous organization, and was somewhat lacking in the hyperaesthesia of rabies. Learned that he had recently been gorging himself with christmas delicacies, and was now very costive, and as there was no satisfactory history af the dog, I at once suspected lyssophobia. The friends and strangers who had come to condole with the parents, and feast on the horror were excluded, and the boy's attention fully engaged in amusing pictures and conversation; the paroxysms were omitted, and, in two hours, the patient overcome by weariness, went to sleep. Next morning, he was still kept secluded and quiet, and two enthusiastic students took up the rôle of keeping his attention constantly engaged on whatever would interest him. The prima viæ was relieved by medicine, and under a course of tonics he quickly recruited, and, at the end of the week, went back to school."

A case occurred in this city, a few weeks ago, which illustrates how a little good sense on the part of a physician may avert a calamity in the same manner so happily effected by Professor Law. A boy of thirteen had harnessed his own and another large dog to a boy's cart. The reins got entangled and the dogs quarreled. The boy went to the assistance of his own dog and was snapped at by the antagonist. He thought little of it, but the boys on the street told him he was doomed to die of hydrophobia. Some grown up—not inmates of a lunatic asylum—but fellow-citizens entitled to vote, joined in this verdict, and the poor boy came home with a face as white as chalk, kissed his mother good-bye, and saying he had to die, disposed of his little possessions. The family physician was called in at about eight o'clock. Instead of pooh-poohing the matter, which, though justifiable, would not have effected his purpose so well, he said: "Well, my boy, I see how it is, you are going through a crisis. If you survive nine o'clock this evening, you are positively safe." The child's attention was thus fixed upon the minute-hand of the clock, after it passed the critical point no more was heard of hydrophobia in *that* house. I cannot suppress the wish that some of the $1000 checks which are flying around in Paris, just now, might drop into the lap of such men as these, who save life, not with loud fan-farronades about microbes (whose existence has not yet been proved), nor who destroy life by creating spurious epidemics through fear and terror, but, who follow out the sensible course, dictated by the knowledge and sympathy of the true physician.

I am aware that a certain philistinism is more or less rampant, just now, in medical literature, which is opposed to every form of criticism. There are minds so organized that they cannot conceive of a man calling attention to the errors of another, unless he have a personal motive. And should one be in the unfortunate position of having played the critic on more than a single occasion, he is accused of having a mania for antagonizing people. Those who are notorious for anonymous attacks on and misrepresentations of their colleagues in the editorial columns of certain medical journals, which seem to have taken the political journal for their model, are often the loudest in denunciation of those who have a sufficient courage of opinion to make their strictures over their signature, and not under the disguise of an editorial inuendoe. I firmly believe that he, who through accident or other means, is in a position to calm public apprehension, has a duty to perform, even at the risk of inviting such criticism as that referred to.

A "Pasteur Institute" has been organized in this city, and is now ready to receive contributions. I believe I am doing that institution no injustice when I say that the more hydrophobia scare .can be created in the community, the better it will flourish. It rests for the present on two foundations: First, on a rabbit brought over from Paris, which had been inoculated by Pasteur, and which died on the way over, or shortly after its arrival. Second, on the allegation that genuine hydrophobia now exists in and near New York City. About the defunct rabbit there is very little to be said; about the alleged existence of hydrophobia, a great deal. In so far as one of the incorporators of the Pasteur institute has contributed to the public alarm on this question, by the published autopsy on one dog, and of an experiment on another, I propose to present for your consideration, not theories, not doubts, not guesses, but facts, which, I believe, will not be gainsaid by any of my medical friends. In so far as I have found it necessary to adduce observations of my own, I do not ask you to accept my bare word for them, but I have brought the very animals to which these observations relate before you. The brains of one or two of them will be removed, and you can examine both the living and the dead subjects, and test the correctness of my conclusions by the aid of the indisputable evidence of your own eyes. Dr. Liautard's experiment is thus stated:

The *Herald* is enabled to give the result of the first successful experiment made in this country according to the Pasteur method, by which undoubted rabies in a dog has been determined. The experiment was brought about by the bite which Miss Morosini received from one of her dogs last month, an event which has already been recorded in the *Herald*. The dog was a household pet and had not previously exhibited any symptoms of rabies. It was killed immediately after it had bitten Miss Morosini, but not before it had bitten several other dogs.

Mr. Morosini was averse to sending his daughter to Paris for treatment by M. Pasteur. At Riverdale it would not be believed that the dog was mad. But Dr. L. D. Buckley, of this city, was promptly communicated with, and he as promptly notified A. F. Liautard, M. D. and V. S. of the American Veterinary College in West Fifty-fourth street. Both gentlemen advised that Miss Morosini should go to Paris at once to see M. Pasteur. Dr. Buckley lost no time in securing the carcass of the dog that had bitten the young lady and sent it to Dr. Liautard. At the Veterinary College a post-mortem was held on the dog, and an experiment made on a living animal by inoculating it with the supposed virus of the dead dog.

A *Herald* reporter yesterday called at the Veterinary College and asked Dr. Liautard, under whose supervision the experiment was made, to make known the results. Dr. Liautard had written a report of the case for the June number of the *American Veterinary Review*, and from the advanced sheets the reporter was kindly permitted to cull the following facts :

Miss Morosini was bitten on April 14. The dog was killed almost at once, and on the same day was sent to the Veterinary College of which Dr. Liautard is chief surgeon. Dr. Buckley requested that a post-mortem should be made as early as convenient, as it was a matter of the greatest importance. The post-mortem was begun without delay, and there were revealed important lesions, congestion of the fauces, empty condition of the intestinal tract which was slightly congested, stomach containing one large bird feather, the kidneys somewhat congested and the bladder empty and retracted and somewhat congested.

"These symptoms were considered sufficient evidence, with the history of the case, to justify a diagnosis of rabies. A report to that effect was made to Dr. Buckley, and it was urgently advised by Dr. Liautard that the injured young lady should as early as possible, go to Paris and place herself under M. Pasteur for treatment. Then it was decided at once to try the cerebral inoculation as carried out by M. Pasteur to confirm the diagnosis.

"The brain and medulla oblongata of the dog having been carefully kept, another dog was secured for inoculation on the following morning. After placing the dog under complete anæsthesia the cranium was trephined a little on one side of the median line. The dura mater was then carefully divided and a little of the medulla of the first dog placed over the cerebrum of the second. The edges were brought together by sutures protected by wadding and collodion, and the animal was placed in a secured kennel for observation. He recovered from the anæsthetic, ate well and the wound healed slowly, presenting nothing apparently abnormal. On April 30th, however, the dog appeared to be more affectionate to those who were caring for him, and also to the house surgeon who watched him.

"On May 1, the sixteenth day after the inoculation, the patient showed the first symptoms of dumb rabies—that is, paralysis of the lower jaw. The mouth was slightly open, the jaw hung down, and abundant saliva flowed from the mouth. Still the dog was very affectionate. The next day the lower jaw became more paralyzed. The animal was weak and tottered in his gait. Paraplegia (or paralysis of both hind legs) now showed itself. His sleep seemed disturbed, his tongue hung from his mouth and he showed no desire to bite. In fact, all the well-known symptoms of rabies were clearly present.

"The diagnosis made at the post-mortem of the first dog, (the one that bit Miss Morisini) was thus confirmed by this experiment and reaffirmed by the post-mortem of the second animal, which died during the night of the 3d. At this autopsy the following lesions were found :—Pharynx highly congested and containing considerable foreign matter, such as hay and straw, the stomach also highly congested. In it were found the same foreign substances seen in the pharynx; the intestines were empty; the bladder empty and firmly retracted in the pelvic cavity."

As the young lady had left New York only on April 22d, and as she could scarcely be expected to reach Paris much before the 2d or 3d of May, there was no time for her to lose in beginning her treatment. Accordingly when the symptoms were sufficiently marked, very early on May 3d, a cablegram was was sent by Dr. Liautard to M. Pasteur, informing him of the result obtained; and on the 5th a cablegram from Europe gave the information that Miss Morosini had received the third inoculation on that day.

"We may therefore conclude," said Dr. Liautard, "that the young lady is now all right. But we could not be sure of that had she delayed her journey to Paris till we found out whether or not the dog that bit her had rabies. This shows the importance of having a Pasteur institute on this continent. M. Pasteur says he will not guarantee a cure if the patient delays coming to him after the thirty-sixth day. The sooner the better. Now, in ordinary cases it will happen that a bitten person will linger till it is put beyond a doubt that the dog is mad. That will take at least fifteen days, and it may have been ten more before the test is begun. There are twenty-five days, and it would probably take the person eleven days to get to M. Pasteur. What chance, then, would there be of a cure? Probably none at all. But with an institute in this country such dangerous delays would not be possible.

"Now, in reference to this experiment, it is noteworthy that symptoms of rabies were manifested in the dog experimented on in sixteen days after inoculation. M. Pasteur says that the symptoms are manifested from the fifteenth day up. Is not this very close?"

Dr. Liautard in further conversation seemed to think that it was possible in the future to have a law compelling all owners of dogs to have their animals inoculated for rabies as a preventative, just as vaccination was compulsory.

As far as Dr. Liautard's diagnosis of rabies, in the dog that bit Miss Morosini is concerned, I have to state that as assistant to Professor Henry Draper, the instructor in physiology, at the N. Y. University, I certainly examined scores of dogs, some of which were purchased from street arabs, and not a few of which I had decoyed within the precincts of the laboratory myself. Not one of the animals showed any sign of rabies. On the contrary, they exhibited that docility and affection, which, under the circumstances, is the most touching characteristic of the animal. There was scarcely one, and, certainly not one young dog, in whose stomach I did not find more or less foreign material, I remember that kite strings, and top pegs were frequent; that coal, ashes, straw, feathers and cotton spools were occasional findings. Shoe leather, pieces of cloth, and, if I remember rightly, a pocket knife, but certainly, some unusual article of metal was found in a pet dog, that had been sent to us owing to the crusade which had been inaugurated against that particular breed just then.*

This became a subject of comment in our laboratory, and I recollect that it was supposed to be a satisfactory solution of the problem that we found intestinal worms in large numbers in those dogs which had the largest and strangest assortment of foreign bodies in their stomachs. A cat which had a similar collection contained three tape worms. The feather in the Riverdale dog, therefore goes for nothing. As to the congested kidneys, congested fauces and empty bladder, I would simply ask you how would the fauces, kidneys and bladder of a man appear, who had been hunted for several hours, by a large crowd, armed with shot-guns and pitchforks?

With regard to the "dumb rabies" which Dr. Liautard thought he had produced in the second dog, every physician familiar with the researches of Schiff, Flourens, Hitzig, Fritsch and Goltz, will recognize in it the ordinary results of experimental and inflammatory disturbance of the brain functions in the dog. According as the irritating injection affects one cortical field or the other, the paralysis will vary, but paraplegia is quite characteristic of meningitis and encephalitis in the dog. In order to show the society that the symptoms of hydrophobia, as Dr. Liautard considers them, can be produced

---

* The *N. Y. Herald*, of that or a later date, headed one column for weeks, with such declarations as "Down with the Bloody Spitz!" "The Spitz Doomed!" "The Spitz must Go!" It was supposed that this particular breed was more disposed to become hydrophobic than any other.

in healthy dogs, by introducing non-rabic foreign substances into their skull cavities, I performed experiments on six dogs, in three relays, that I might be certain to have some of them alive to-day.

*Experiment A*, May 29th.—Mongrel bull dog, was etherized at 1.30, a longitudinal incision made parallel to the median line, the periosteum raised by a crucial incision, the trephine applied, and as the diploe was unusually loose the instrument had to be applied a second time to remove the inner table. Considerable hemorrhage occurred from the diploe, and probably from a meningeal vein, wounded in slitting the dura. An incision one half a centimetre in length was made in the latter, and about six cubic millimetres of the spinal cord of a healthy calf, slaughtered about twenty-eight hours before was introduced on the brain surface. Some hernia cerebri ensued. The wound was united by four sutures, an opening being left for drainage. The operation, including the etherization, lasted about six minutes. The dog was put in a stall, and fifteen minutes later was on his feet and tottering around.

June 2d.—The animal is feeding and drinking, again ; it had not fed for a day or so after the operation. There is a slight droop of the left upper eyelid ; the eyes appear dull. There is manifest paralysis of the hind legs, the tail is sometimes drawn in between them. The disposition of the poor animal is exceedingly friendly, wagging his tail feebly, and crawling forward and fawning as soon as the door is opened. There is considerable fever and the eyes are dull ; there is ptosis. The wound is granulating and discharging healthy pus.

June 5th.—Animal is not feeding so well, less feverish, very much emaciated, the eyes are brighter, the paralysis of the hind legs does not manifest itself in the straddling gait noted a few days ago, but in a sort of wabble. On getting out into the court-yard, the animal began swallowing horse dung and was driven back to its stall in consequence. The wound is not looking so well ; it is dressed with carbolated vaseline.

June 6th.—Disposition to us unchanged, quarrelled over the milk with dog C.

June 9th.—The paraplegie gait is very marked. The animal seems to be acting impulsively at times. It attempted to swallow a dry drum-stick of a fowl. It would have again eaten horse-dung had it not been prevented.

*Experiment B.*—A second dog, a mongrel spitz, was operated on immediately after the former. A T-shaped incision was made over the right temporal muscle, the periostenum scraped as before, and a button of bone removed. A small opening had been made by the center pin in the dura, and through this some of the same material used in the last experiment was introduced in a comminuted form. There was not much hemorrhage in this case. The wound was closed by sutures, and the animal placed in another stall.

June 2d.—The animal has been doing well; it is kept on low diet; there is a little discharge from the lower angle of the wound of a benign character. It was noted to be rather an aggressive animal before the operation, and had, together with the other dog (A), barked at visitors, and made demonstrations between attack and fawning which all small dogs, particularly of that breed, are apt to make. It is to-day quite a different animal, fawning, wagging its tail, and clings to any one who will give it a friendly word. In running, the hind legs are not quite as steady as the fore legs; in stepping among the round stones with which the court is paved, that lack of skill, so well described by Hitzig, is noticed—the animal sways considerably, and crouches with the hind-quarters, but is able to rise on its hind legs.

June 5th.—There is considerable improvement in motion. The greater part of the day the animal lies on its side, paying no attention to the door when opened, or to those who walk around and close to it. The wound is doing very well.

June 9th.—The paraplegia is more marked; the animal is unable to jump down a distance of one or two feet, but lets itself down on the fore-feet and then slides down with the rest of its body. It has undergone an entire change in demeanor, being shy, avoiding visitors, and instead of coming when called, as it did June 5th, crouching and concealing itself. At the same time, the animal appears apathetic.

*Experiment C*, June 2.—A large mongrel black-and-tan etherized, terebration performed to the right of the median-line about one inch. The instrument being defective wounded the brain, and some brain matter was found adhering to it. A hypodermic needle was then introduced through the opening and twenty drops of an emulsion of a calf's cord slowly injected into the brain wound. Some of this escaped. The animal was allowed to recover in a stall. The slight opening in the skin was not sutured.

June 5th.—The wound is scarcely noticeable. The animal is very feeble in its limbs, although in good general health. It can scarcely step sideways without going down on the hind legs, and a slight ataxia is noted in the fore. Both paralytic and ataxic symptoms appear more marked on the left side. The animal appears to be stupid and dull.

June 6th.—The same feebleness in the hind limbs is noted in a marked degree. The animal seems a little brighter

June 9th.—The animal is very stupid; runs against objects; does not avoid obstacles, and exhibits decided *manége* movement, running in circles to the right. On being roused, when lying down, which is the animal's usual position, it rolls over and frequently slips with its hind legs which tremble a great deal. At night this dog howls a great deal, and growls at people entering the court at all times. There is a question as to whether it is able to see well; but owing to its stupidity, it is difficult to settle this point satisfactorily.

*Experiment D.*—Mongrel collie of a vicious disposition, same operation as in C, an emulsion was prepared from the pons and cerebellum of a gentleman who died of hydrophobia on May 14th, and whose brain and cervical cord I obtained through the courtesy of Dr. Paul H. Kretschmar, of Brooklyn. This was injected into the parietal region of the brain, and—by calculation—into the thalamo crural region. While the animal was being placed in the stall he managed to escape the attendant, and ran around, avoiding those who attempted to block his passage or catch him with considerable skill. He seemed to be acting under some impulse which kept him "on the go." This dog acted differently from the others under the ether, and his cursorial exhibition was, probably, but the result of intoxication; it closely resembled, however, the result of experimental irritation of the so-called cursorial center in rabbits as described by Nothnagel.

June 5th.—This animal has been "on the go" ever since, but gradually becoming quieter. It runs around actively; there is a little ataxia of the hind legs, and when it runs up to a bar it comes to a halt, appears puzzled, but although gross motor power is unimpaired it neither passes over nor under it, but goes back and around, contrary to its previous habit.

June 6th.—Lies in a corner of the stall with its head hidden in a crevice, and growls and snarls viciously as I enter the stall. On being taken out and tied it makes no effort to bite the stable help, but flies at me and barks furiously. Dr. Hamill found no difficulty in controlling it. There is some question as to whether there is some weakness of the hind-quarters. It is, to-day, the liveliest of the four dogs, and feeds best.

June 9th.—It is not as savage to-day, the pupils are very narrow and the expression appears dull. The sexual instinct is manifested unmistakably to-day.

The emulsion injected into this dog's brain, which was injured in the same place, and to the same extent as that of experiment C, consisted of human material preserved in glycerin and salt, of human material dried and of brain from a rabbit which had been innoculated with the above when fresh, but died on the eighth day from *fungus cerebri*.

*Experiment E.*—Large yellow dog etherized, trephined; a slit made in the dura, and a piece of common yellow soap of the size of fifteen cubic millimetres introduced between the dura and pia. There was considerable hemorrhage from the diploe, and it was subsequently suspected by one of my assistants that hemorrhage occurred from a superficial artery, whose wound was undiscovered owing to compression during the operation.

June 9th.—Severe constitutional symptoms have been shown by this dog. The jaw droops, and the tongue is often protruded between the teeth. It runs around aimlessly; arrived at a running hydrant, it stands still, as if pensive, but does not drink. There is considerable palsy of the hind quarters, the legs straddling so much that the body nearly touches the ground. There is great sensitiveness of the surface of the body.

*Experiment F.*—Young mongrel poodle etherized, terebration performed, and ten drops of stale horse's urine injected into the brain substance of the posterior region. This animal had a glandular and suppurating swelling in the inferior nuchal region.

June 9th.—The animal did not eat after the first day following the operation though having recovered nearly entirely from the immediate effects. It became paraplegic, moaned a great deal, and developed inability to swallow, though attempting to drink, yesterday. To-day, at noon, it died. An autopsy showed, singularly enough, no reaction at the site of the injection, but the basilar meninges were filled with a greenish-yellow gelatinous exudation, and the ventricles with a turbid purulent serum. The appearances being those of acute meningitis. When the spinal canal was divided a large amount of purulent serum with floeculi of fibrin ran out. So intense was the distension of the ventricles, that the gyri of the convexity had been pressed nearly flat against the dura, and this against the cranium.

You may decide for yourselves the following:

1. Is there any symptom of hydrophobia related of Dr. Liautard's animal, which is not present in these dogs inoculated with the spinal cord of a calf, with soap and with decayed fluids?

2. Are the mental disturbances resembling those of dumb rabies—so-called—not even much greater in the dogs to-night exhibited than appears in the account of Dr. Liautard's dog?

3. Would it not have been well to have performed such experiments as those whose result you see to-night, at the same time, or before attempting to make of the inoculation of the brain a test of hydrophobia, and alarming the public?

4. Was it in view of the bad effect which the knowledge that the dog who bit a person was mad has on that person's mind, wise to make this announcement in such a shape that it must reach him or her?

5. What reason had Dr. Liautard, or his house surgeon,* for omitting an examination of the brain in the case cited, or—if they made it—for failing to publish its morbid appearances?

I have been thus lengthy in detailing the faulty nature of the evidence on which an epidemic of hydrophobia may be announced, and, in demonstrating that, mere terror and expectant attention, are the chief factors in the spread of the disease, because the practical problem with which preventive legal medicine has to deal is not so much the prevention of a genuine as of a more or less artificial disorder. Inasmuch as the same measures which will check the spread of the real disease—if it exist—will also, and as effectually check the ravages of that terror-psychosis, known as lyssophobia, we may deal with both together.

---

* The description in the *N. Y. Herald* is attributed to Dr. Liautard; it is reproduced from advance sheets of the "*American Veterinary Review*," of June, 1886; but these appear under the authorship of James A. Walrath, D.V.S., house surgeon, who opens the article with a becoming reference, as follows: "A dog had bitten Mrs. M——, also biting other dogs, and had been killed, a careful post-mortem examination following, Dr. L's (Liautard's) *expert judgment and his immediate attention* to the case were *urgently solicited.*

The bite of any animal, or even of men, suffering from a febrile disorder, from excitement, tetanus, or from blood-poisoning. is more or less dangerous. Undoubtedly many of the sequalæ of such bites have been summed up under the general caption of hydrophobia.* Whatever theory we adopt, it is the duty of the community to protect its members not only against the more or less venomous bites of such animals, but also against intrinsically harmless bites. Historically speaking, the system of muzzling dogs has been found so efficacious that it is simply surprising that it has not been universally adopted. During the years 1845–1853, inclusive, 278 rabid dogs were entered at the Berlin Veterinary College, an average of about thirty cases a year. In the year 1854, an order for the muzzling of all dogs was rigidly enforced by the police. The result was that in this year, there were but four cases; in each of the two following years, only one case; and in the five years from 1857 to 1861, inclusive, not a single one.† Gibier states that, when in order to pursue the researches for which he received a reward from the French Academy of Medicine, he applied at the Berlin Veterinary School to have a mad dog, he was informed that there had not been a case for three years.‡

The plan of muzzling dogs has been adopted in England. Everything, of course, depends on the manner in which the law is enforced. If, as is only too likely in our own community, the persons entrusted with its execution be selected from the worst class of ruffians, and their pay be made dependent on the number of dogs they can steal from their owners, and the amount of blackmail they can levy through such acts, the public will become disgusted with the law, and those evading it will be supported, instead of being condemned, by public opinion.

Another important precaution which has been almost uniformly neglected on this side of the Atlantic, is the proper ob-

---

* Planat (*Annales Medico Psychologiques*, 1884, p. 383), in all seriousness, publishes a case where a pet epileptic cat bit a husband and wife, who thenceforth became epileptic, as one of transmission by biting! It seems superfluous to say that the microbe (?) of epilepsy has not been described as yet by the most enthusiastic bacteriologist. The case cited proves what nonsense may exist under the garb of science.

† Law, op. cit., p. 890.

*British Medical Journal*, January, 1886.

servation of suspicious dogs. There is no evidence so good as that obtained from the living subject of rabies. In addition, much suffering, and indeed many deaths might be prevented by the discovery that a dog who has bitten is not mad; and that can be determined with certainty, only by keeping the animal alive.

The instructions of the Governmental Councillors, in Germany, in regard to autopsies on animals suspected of rabies, read as follows:

"The contents of the stomach and intestines, and the condition of their mucous membranes are to be determined above all. Then the condition of the spleen, kidneys and liver is to be described. The pharynx, tonsils, trachea, lungs and heart are next to be examined. Along with this the condition of the blood, particularly with regard to its coagulability, is to be carefully noted: finally, the cranium should be opened and the brain examined.

Gscheidlin suggests that this order be so amplified as to direct also the making of inoculations, and, if these cannot be made by the veterinarian in charge, the material to be sent to some designated expert or institution. It is to be understood that only responsible persons be intrusted with such experiments, as animals infected with septic material may do injury if improperly discharged.*

With regard to the recognition of a Pasteur institute as a factor in the legal enactment of measures to prevent hydrophobia, I find language in one of the leading medical periodicals of the world, which so thoroughly voices my own opinion that I cannot do better than cite it.

†"Attention has been repeatedly directed in this journal to the fact that the method of Pasteur can not be unreservedly accepted as an established one, and that the foundation of Pasteur Institutes is premature until the method of preventive inoculation be placed on an absolutely fixed basis, which in view of the large number of experiments on animals, and inoculations made daily, in Pasteur's laboratory, will presumably be effected in a not unduly remote period. It were high time, indeed, that we left behind us the stage of newspaper reports, which for some months have furnished nothing new be-

* It was stated in the daily papers that a person had been bitten by a hydrophobic rat, which a medical student had inoculated. But, possibly, as he was reported to have resorted to the Mecca of all true and false hydrophobia believers, this case may have been manufactured out of the same cloth as the lessons which Duke Charles, of Bavaria *never intended* to take of Pasteur, and the Commission which the Austrian Government did *not* send!

† Guttman in *Deutsche Medizinische Wochenschrift*, April 29th, 1886, p. 203.

yond the daily increasing number of those inoculated, and the deaths, and replaced the present monopolization of the matter by the cooperation and critical proof tests of competent investigators."

In concluding these remarks, I have a task of gratitude to perform in acknowledging my indebtedness to those, without whose aid, these experiments would have been impossible. Dr. James Hamill placed the veterinary infirmary of Atcheson & Hamill at my disposal, and supplied me not only with abundant material, but made a number of observations in my absence, which completed my records. To our fellow members, Drs. N. E. Brill, F. H. McGuire, and R. Mollenhauer, I am likewise indebted for assistance rendered in the necessary operations.

